# Effect of Cu Content on the Alloy Tensile Properties of Al-Cu Based Alloys Tested at 25 °C and 250 °C: Application of the Concept of Quality Index

**DOI:** 10.3390/ma16041400

**Published:** 2023-02-07

**Authors:** Abram Girgis, Ehab Samuel, Agnes M. Samuel, Victor Songmene, Fawzy H. Samuel

**Affiliations:** 1 Département des Sciences Appliquées, Université du Québec à Chicoutimi Québec, Chicoutimi, QC G7H 2B1, Canada; 2 Department of Mechanical Engineering, École de Technologie Supérieure (ÉTS), Montréal, QC H3C 1K3, Canada

**Keywords:** Al-Cu alloys, heat treatment, tensile testing, quality index, FESEM

## Abstract

The present work was performed on three versions of a newly developed alloy coded T200 containing 6.5% Cu, 0.1% Fe, 0.45% Mg, and 0.18% Zr in addition to A319 and A356 alloys (grain refined and Sr-modified). Tensile bars were subjected to 13 different heat treatments prior to testing at either 25 °C or 250 °C. The tensile data were analyzed using the quality index method. The results obtained showed that, due to the high copper content in the T200 alloy coupled with proper grain refining, the alloy possesses the highest quality as well as improved resistance to softening when tested at 250 °C among the five alloys. The results also demonstrate the best heat treatment condition to maximize the use of the T200 alloy for automotive applications. Grain-refined alloy B, treated in the T6 temper and tested at 250 °C, exhibited the best combination of the four tensile parameters, i.e., UTS, YS, %El, and Q-values: 308 MPa, 304 MPa, 2.3%, and 352 MPa, respectively, which are comparable with those obtained from the 356 alloy: 309 MPa, 305 MPa, 2.8%, and 375 MPa in the same order.

## 1. Introduction

The specific qualities of foundry aluminum alloys are, in particular, good castability, low melting temperatures, absence of hot cracking, and good distribution of porosities due to shrinkage during solidification [1]. Casting aluminum-silicon (Al-Si) alloys are the most widely used due to their very good castability and good corrosion resistance. The mechanical properties and structural characteristics of foundry Al-Si alloys are markedly affected by their composition in alloyed elements and by the heat treatments to which they are subjected [2]. The metallurgical factors specific to Al-Si base casting alloys are considered to achieve optimum service performance in their applications. Alloying elements, depending on their composition in the various alloys, generally contribute to increasing the ultimate tensile stress and the elastic limit. These modifications generally lead to a reduction in the strain at break. Indeed, the higher the ultimate tensile stress and the elastic limit, the lower the strain at break. Therefore, trade-offs must be constantly made between the need to obtain high values of ultimate tensile stress and yield strength on the one hand and obtaining ultimate strain or ductility on the other hand. However, the situation is altered by the presence of alloyed elements in some Al-Si eutectic alloys, including intermetallic compounds that are more fragile than silicon particles. Therefore, the failure of the alloy is initiated from the intermetallic compounds. It has been established that, in the eutectic binary Al-Si alloys, the cracks originate in the silicon particles, in particular at the boundaries of the dendrite cells. These cracks then spread to the aluminum matrix and initiate ductile fracture [1,2,3].

The addition of copper (Cu) to Al-based alloys produces a significant increase in HV hardness and ultimate tensile stress, or ultimate stress of a typical alloy, or a 15% increment of strengthening. Moreover, the role of copper in the reinforcement by precipitation of the resistance of Al-Mg-Si alloys has been studied by many authors. Pashley et al. [3] reported that the presence of copper leads to the formation of a precipitate with a much finer structure in samples subjected to artificial aging and, thus, leads to better mechanical resistance following this heat treatment [4].

The most significant effect of copper is at the third stage of the precipitation sequence. For Al-Mg-Si alloys, this third stage is the β′ phase, which has a rod shape with a hexagonal structure. With the addition of copper to Al-Mg-Si alloys, lath-shaped precipitates are observed; their quantity increases with an increase in the copper composition. More recently, Chakrabarti et al. 5 suggested that this lath-shaped phase could be a precursor of the Q phase, named Q′. The addition of copper has also been reported as an element that reduces the negative effects of natural aging [5,6,7]. Recent studies by Gökçe et al. [8,9] on Al-Cu and Al-Cu-Mg alloys revealed that high-strength, Al-based powder metallurgy alloys were developed with good microstructures from the premixed elemental components with an increase in the strength of the base Al powder by 5-times from 84 MPa to 466 MPa.

The concept of a quality index was first proposed by Drouzy et al. [10,11,12] for Al-Si-Mg (356 alloys). The quality index relates the quality of castings with their mechanical strength and is expressed with the following equation:

Q = UTS + d log (%El)(1)

where Q = quality index (MPa); UTS (ultimate tensile strength) in MPa; %El = % elongation to fracture; and d is a constant, whereas YS (probable yield strength) is defined as:

YS = a UTS − b log (%El) + c(2)

The probable yield strength (YS) is controlled by hardening agents, such as Mg and Cu [13].

Previously, the authors presented their findings on the hot tearing and microstructure of the recently developed Al-Cu-based alloy, which is a modified version of the 208 alloy with a higher Cu content and no Si, in comparison with A356 and A319 cast alloys [14]. The present investigation focuses on the microstructural characterization, tensile properties, and quality index of these three types of alloys following 13 different heat treatments and two testing temperatures: 25 °C and 250 °C. In addition, the results will be compared with those obtained from two commercial alloys, i.e., 319 and 356 alloys, that are widely used in automotive applications. The study will also include a detailed analysis of the effect of all variables on the quality index in order to determine the optimum composition and heat treatment. Another direction considered will be the contribution of each of these parameters to the tensile properties of the base alloy (coded A in the present study). Generating all these data will help in enhancing the performance and determining the exact composition of the newly developed Al-Cu-based alloy that would provide tensile properties close to those of the currently used commercial alloys.

## 2. Experimental Procedure

The chemical composition of the base alloy T200 is shown in Table 1, whereas Table 2 lists the final compositions. Two more versions of the T200 alloy were prepared through the additions of 0.15 wt% Ti + 0.15 wt% Zr and 0.15 wt% Ti + 0.15 wt% Zr + 0.5 wt% Ag using Al-5%, Ti-1% B, and Al-15% Zr master alloys; Ag was added as a pure metal (999.99%). The A319 and A356 alloys were grain refined using 0.15 wt% Ti and modified with 200 ppm Sr using Al-5%, Ti-1% B, and Al-10% Sr master alloys, respectively. The used alloys were termed A, B, C, D, and E (Table 1). Table 2 lists the final compositions.

All alloys were received in the form of ingots and melted in a 60-kg capacity SiC crucible using an electrical-resistance furnace (Pyradia, Montreal, QC, Canada). This furnace is equipped with a rotary degassing impeller. The melting temperature was varied between 830 °C and 750 °C depending on the alloy’s final composition. Prior to casting, the temperatures of all melts were approximately 750 °C. However, the temperature in the pouring ladle was approximately 720 °C. The metallic ladle was also coated with boron nitride and preheated at 350 °C prior to transferring the liquid metal to the mold. For each alloy composition, the specified alloying elements were added using calculated amounts of the corresponding master alloys to obtain the desired level of addition.

The molten metal was degassed for approximately 15 min using pure, dry argon gas injected into the melt at a constant rate of 20 m^3^/h employing the graphite impeller (rotating at ~120 rpm). In order to ascertain the exact chemical composition, three samplings for the chemical analysis were taken at different times during the casting process for each alloy melt. These samplings were taken at the beginning, at the middle, and at the end of each casting process. The melt was poured into an ASTM B-108 permanent mold (fabricated in-house using ASTM B-108 standard specifications) preheated to 450 °C (to drive out moisture) to prepare the test bars for tensile testing (solidification rate of ~8 °C/s) [15].

The as-cast bars (570 tensile bars divided into sets of five bars each) were subjected to different heat treatments to enhance their mechanical properties. The different heat treatment conditions, incorporating solution heat treatment (SHT), quenching, and aging (T6 and T7 tempers), that were used for this study are given in Table 3, which gives the heat treatment details for Alloys A, B C, D, and E.

- All solution heat treatments (SHT) for the T200 alloys were carried out at 520 °C.
- All solution heat treatments (SHT) for the A319 alloy (coded D alloy) were carried out at 500 °C for only 8 h.
- All solution heat treatments (SHT) for the A356 alloy (coded E alloy) were carried out at 540 °C for only 8 h.
- Water quenching was done using warm water (~60 °C).

Tensile testing at ambient temperature was carried out on half of the total number of test bars obtained for all the alloys and all conditions (as-cast and heat-treated). An MTS Servo hydraulic mechanical testing machine (MTS Systems Corporation, Eden Prairie, MN, USA) was used to carry out the tensile testing at a strain rate of 4 × 10^−4^ s^−1^. Tensile testing at 250 °C was carried out on the other half of the total number of test bars (265 bars or 53 packets) for all the alloys/conditions studied. In this case, the testing was carried out employing an Instron Universal Mechanical Testing machine (Instron®, Norwood, MA, USA) using the same strain rate of 4 × 10^−4^ s^−1^ as in the ambient temperature case. In both cases, a data acquisition system attached to the machine provided the tensile properties in terms of ultimate tensile strength (UTS), yield strength at 0.2% offset strain (YS), and the percentage elongation to fracture (%El).

The samples for scanning electron microscopic (SEM) examination were prepared from the tensile-tested specimens by sectioning them 1 cm immediately below the fracture surface and mounting them carefully for subsequent fracture surface examination. The fracture surface of the selected samples was examined using the same JEOL 840A scanning electron microscope (JEOL, USA Inc., Peabody, MA, USA). The fracture surface analysis aimed to investigate the nature of the fracture for the selected samples and identify the main source of cracking and fracture for these alloys. The polished surfaces were ion milled for 20 min prior to examination.

## 3. Results and Discussion

### 3.1. Microstructural Characterization (Solidification Rate ~0.8 °C/s)

**Figure 1:**
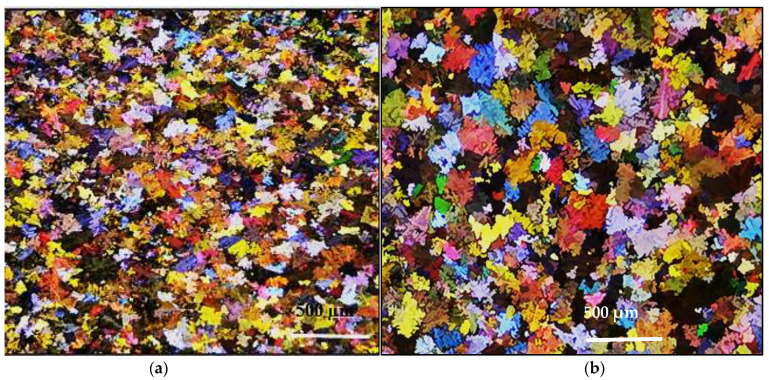
Macrostructure of grain size distribution in: (**a**) alloy A and (**b**) alloy D in the as-cast condition.

Figure 1 shows the distribution of grain size in the present T200 alloy and the commercial 319 alloy. It is evident that the combined addition of Zr + Ti is more effective in refining the alloy grains than using TiBor alone.

**Figure 2:**
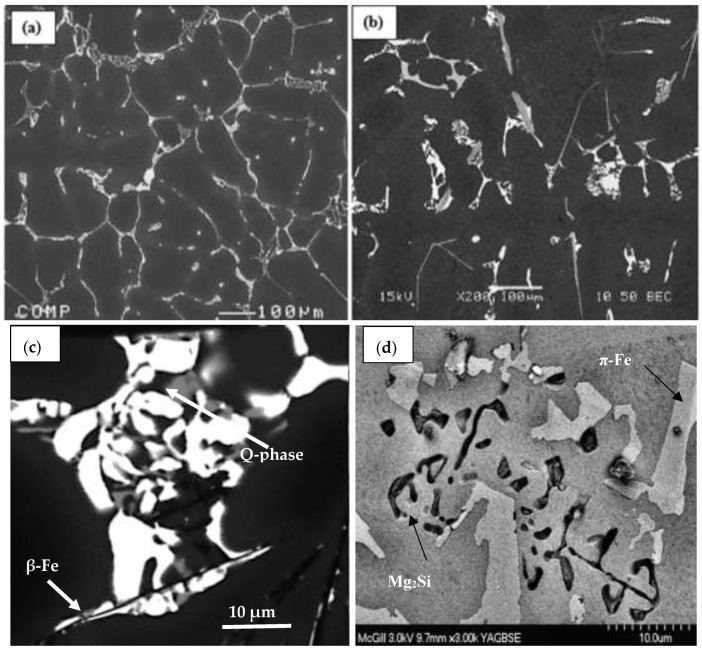

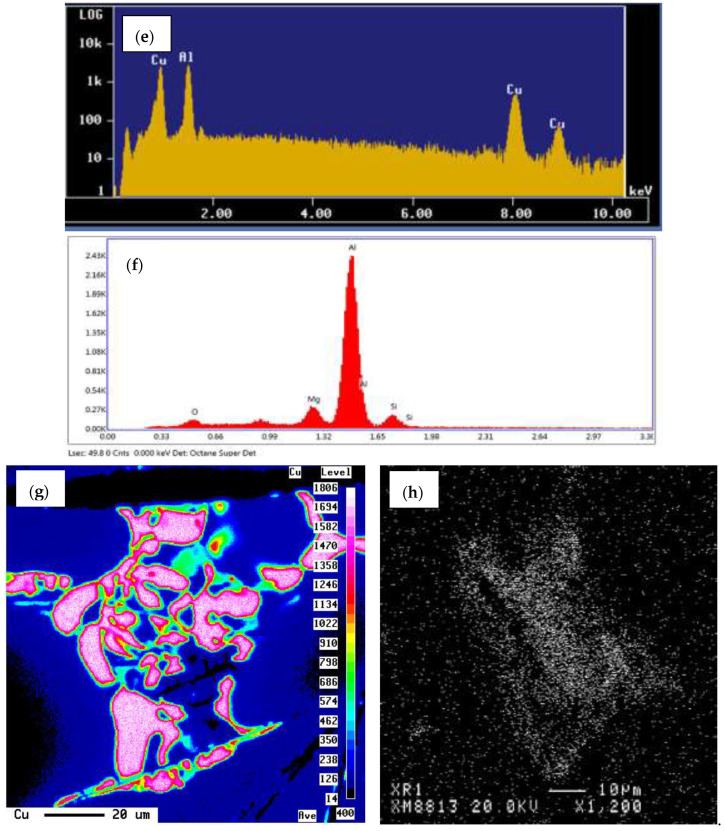
Backscattered electron images of the three base alloys in the as-received condition: (**a**) alloy A; (**b**) alloy D; (**c**) alloy D high magnification; (**d**) alloy E; (**e**,**f**) EDS spectra corresponding to Al_2_Cu and Mg_2_Si, respectively; (**g**,**h**) X-ray images of Cu and Mg distribution in (**c**) and (**d**), respectively.

**Figure 3:**
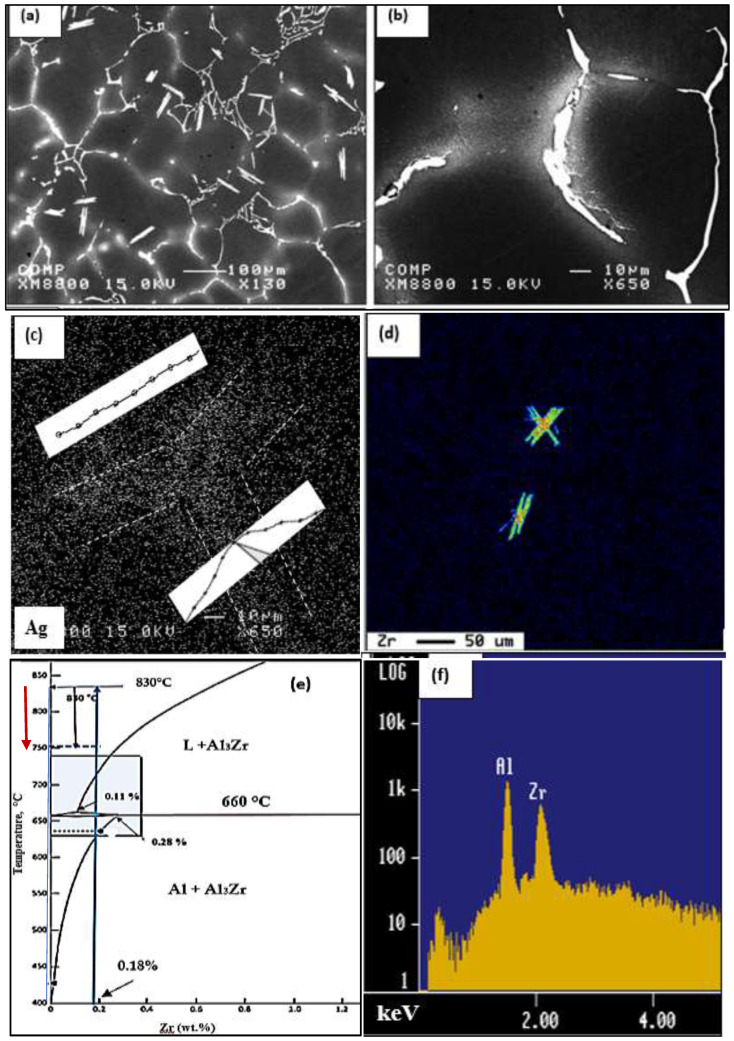
Phase precipitation in alloy C in the as-cast condition: (**a**) backscattered electron image of alloy C; (**b**) high magnification of (**a**) showing segregation of Ag towards the alloy grain boundaries as confirmed with the x-ray image in (**c**); (**d**) X-ray image of Zr precipitated particles; (**e**) Al-Zr binary diagram. The blue arrow points to the melting temperature, whereas the red arrow indicates the pouring temperature where Al_3_Zr phase particles in (**a**,**d**) were precipitated; (**f**) EDS spectrum corresponding to the Al_3_Zr particle in (**d**). The inset in (**b**) reveals the peak distribution between the white lines.

Figure 2 illustrates the microstructure of the as-received alloys revealing the different phases and their relative volume fractions. It is evident that, due to the high copper content in alloy A, the number of precipitated phases (mostly Al_2_Cu) is markedly higher than those in alloy D associated with small particles of the Q-phase. Since alloy E contains only Si and Mg, Mg_2_Si is the main precipitated phase, as shown in Figure 2d, along with fine β-Al_5_FeSi platelets and π-FeMg_3_Si_6_Al_8_. Figure 3 reveals the precipitation of Al_3_Zr within the aluminum grains, whereas Ag was segregated towards the grain boundaries in alloy C. According to the Al-Zr binary diagram shown in Figure 3e [16], the liquidus point of 0.3% Zr is approximately 750 °C. The melting temperature was 830 °C, sufficient to dissolve most of the added Zr. During the period of reducing the melt temperature to 750 °C prior to casting, some of the Al_3_Zr might have precipitated in the form shown in Figure 3a.

### 3.2. Tensile Properties (Solidification Rate ~8 °C/s)

#### 3.2.1. Testing Temperature 25 °C

**Figure 4:**
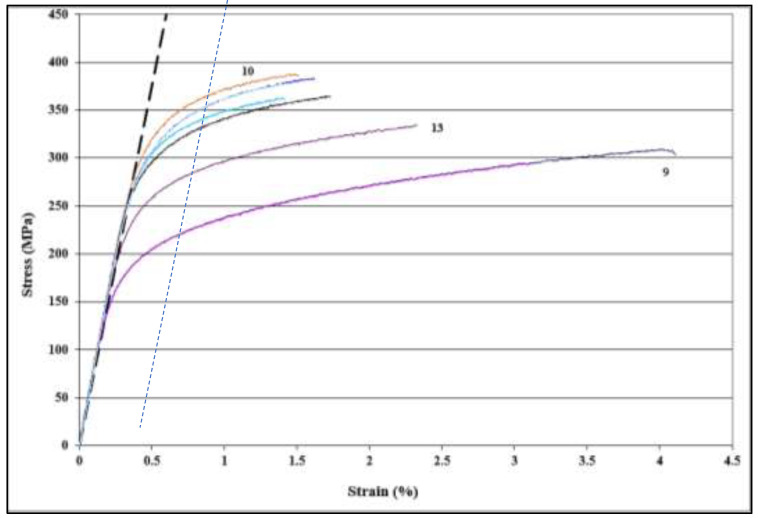
Stress-strain diagrams obtained from alloy B at room temperature following different heat treatment conditions. The broken blue line indicates 0.2% proof strain. The colors of the curves correspond to the heat treatment conditions as listed in Table 3 – of interest to note are the curves for treatments 10, 13 and 9.

**Figure 5:**
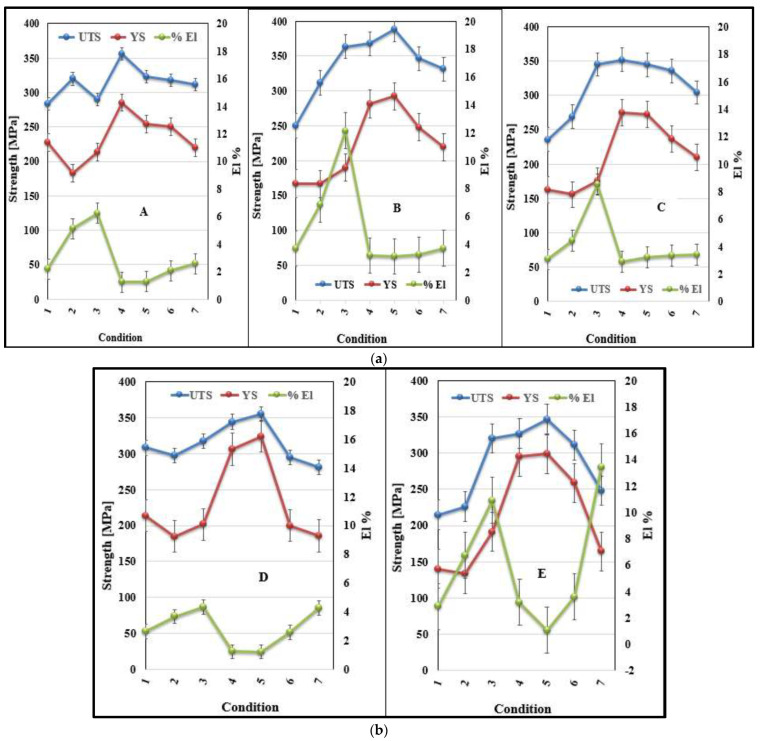
Variation in the tensile properties as a function of the heat treatment and alloy composition: (**a**) three versions of the T200 alloy; (**b**) commercial alloys.

**Figure 6:**
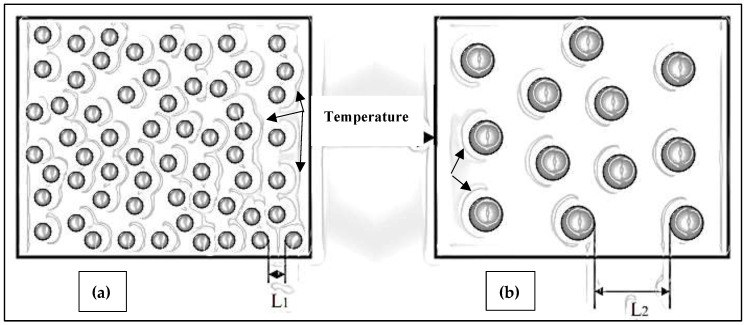
Schematic representation showing the influence of increasing the aging temperature on the size, density, and inter-particle spacing of the hardening precipitates: (**a**) at a low aging temperature and (**b**) at a high aging temperature. (L1 and L2 indicate the inter-particle spacing in each case.). The black arrows represent the dislocations motion through the precipitates.

Figure 4 shows the stress-strain curves produced from alloy B tested at 25 °C following different heat treatments. Examination of these curves shows that they all reveal the same Young’s modulus. However, there is no marked change in the rate of work hardening when going from one condition to another due to the large amount of alloying elements in the base alloy (approximately 8%). As may be seen from Figure 5, all three tensile parameters of alloy C are slightly inferior to those obtained from alloy B, which may be due to the entrapment of some oxides during the course of casting. The beneficial effect of the addition of 0.5% Ag appeared not in improving the alloy strength, but rather in enhancing the alloy resistance to softening during the heat treatment, in particular aging in the T7 process compared to other alloys. Prolonged aging for 100 h at 180 °C resulted in the coarsening of the Al_2_Cu particles, which explains the drop in the alloy strength as presented in Figure 5b. Considering treatments 8 to 13, it is evident from Figure 6 that alloy B exhibited superior tensile strength compared to that obtained from the A319 and A356 alloys.

**Figure 7:**
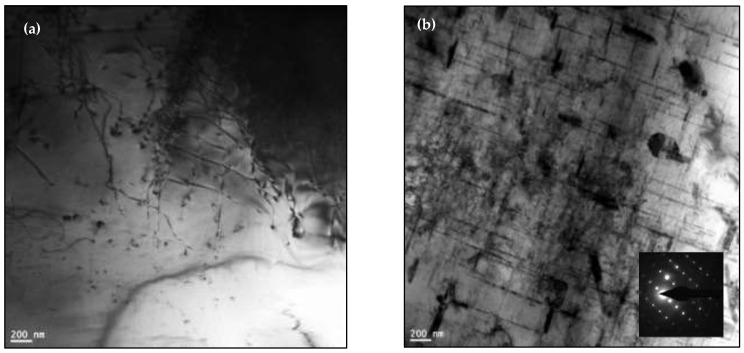
Brightfield TEM images of dislocation-particles in (**a**) T4 and (**b**) T6 conditions, revealing the significant increase in dislocation density—same zone axis [17].

**Figure 8:**
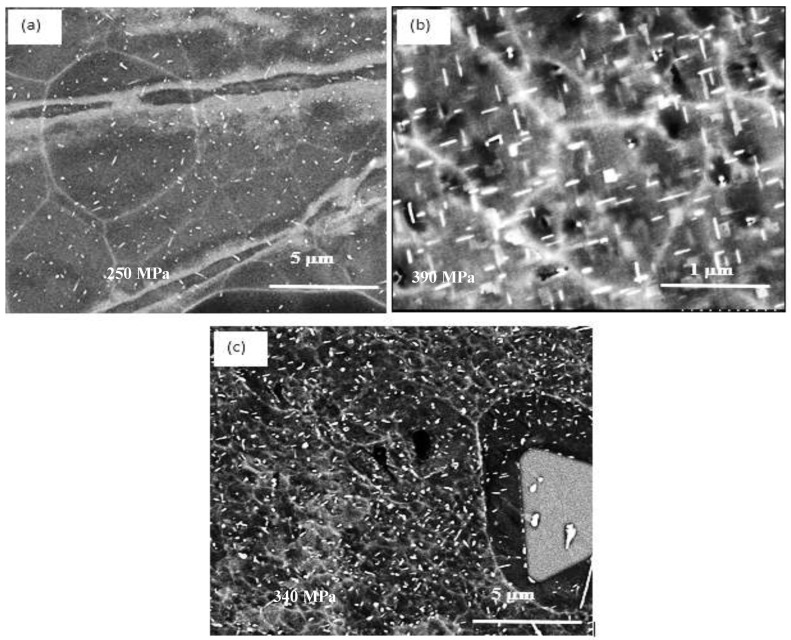
Variation in precipitates density and morphology as a function of applied heat treatment in alloy B: (**a**) as cast (condition 1); (**b**) T6 (condition 5); (**c**) T7 (condition 7)—Numbers in each image indicate UTS values.

Figure 6 is a schematic diagram showing the effect of the heat treatment on the dislocation motion and particle density of the alloy strength when going from the T6 to T7 condition. Abdelaziz et al. [17] investigated the dislocation-particles interaction as a function of heat treatment in the 354 alloy, as depicted in Figure 7, using transmission electron microscopy. In the case of the present alloys, Figure 8 exhibits the variation in the precipitates as a function of the applied heat treatment.

**Figure 9:**
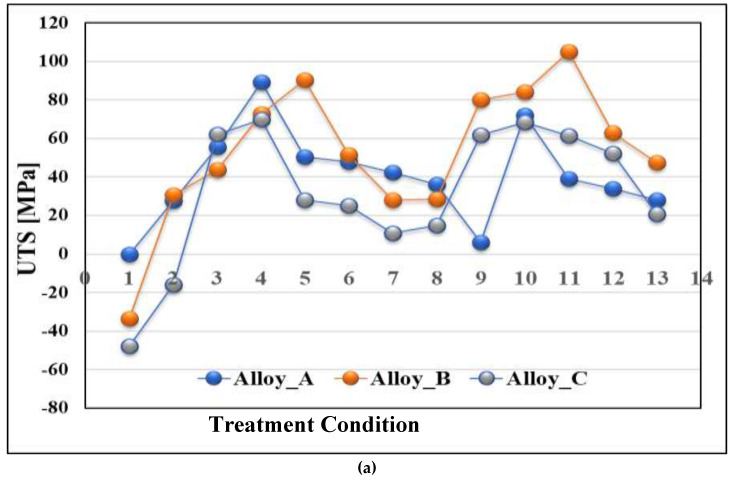

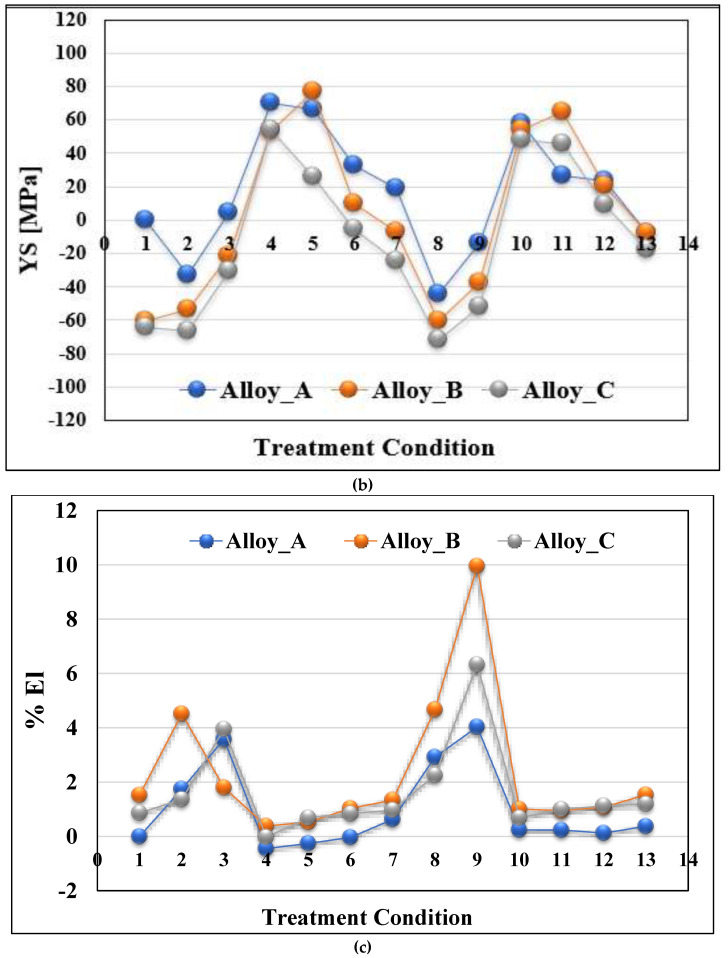
Contribution of the alloying elements and heat treatments to the tensile properties of alloy A in the as-cast condition: (**a**) UTS; (**b**) YS; (**c**) % elongation to fracture.

Table 4 lists the tensile properties of alloy A in the as-cast condition. Based on these values, the plots in Figure 9 show the tensile properties for a given alloy composition/heat treatment condition (P = property) in terms of ∆P values where ∆P represents the difference in P with reference to the same property for the A alloy in the as-cast condition. Thus,

∆P = P (at a given condition/alloy composition) − P (alloy A-as cast)(3)

These plots, therefore, depict the contribution of the added elements as well as the heat treatment processes to the tensile properties of the as-cast base alloy A. It should be borne in mind that negative values mean that the alloy properties in the as-cast condition are superior to those obtained after heat treatment.

The main observations that may be noted/summarized from Figure 9 are as follows:

From Figure 9a with respect to the UTS tensile property, it is seen that:

1. Alloys B and C show a decrease in UTS levels by approximately 40–50 MPa in the as-cast condition.
2. The maximum contribution was obtained for all three alloys when heat treated in the conditions 4, 5, 10, and 11 with alloy B achieving slightly higher values (approximately 20 MPa) than the other alloys, reaching approximately 110 MPa above that shown in Table 4 for the as-cast alloy A.
3. Treatment 8 resulted in the lowest contribution to the UTS level: 40 MPa (alloy A), 30 MPa (alloy B), and nil for alloy C containing 0.5% Ag.
4. Treatment 4 offered an intermediate contribution almost half-way between treatments 8 and 1.

With respect to the YS tensile property, Figure 9b reveals that:

1. The maximum contribution to the YS of the as-cast alloy A was achieved when treatments 4, 5, 10, and 11 were applied to the three alloys, i.e., A, B, and C, with a value of approximately 80 MPa.
2. In this case, also, treatment 8 contributed negatively to the YS (40–60 MPa) with alloy C exhibiting the minimum YS of the three alloys.
3. Treatment 13 brought all alloys to almost none.
4. Treatments 1–3 had the same effects on the YS as Treatment 8.

For the percentage elongation to fracture, the following observations were noted from Figure 9c:

1. The plot showed two explicit positive peaks, one after treatment 2 (4%) and the second following treatment 9, where each alloy contributed differently: 10% (alloy A), 6% (alloy C), and 4% (alloy A).
2. The remaining heat treatments exhibited contributions as little as 1%.

#### 3.2.2. Testing Temperature 250 °C

**Figure 10:**
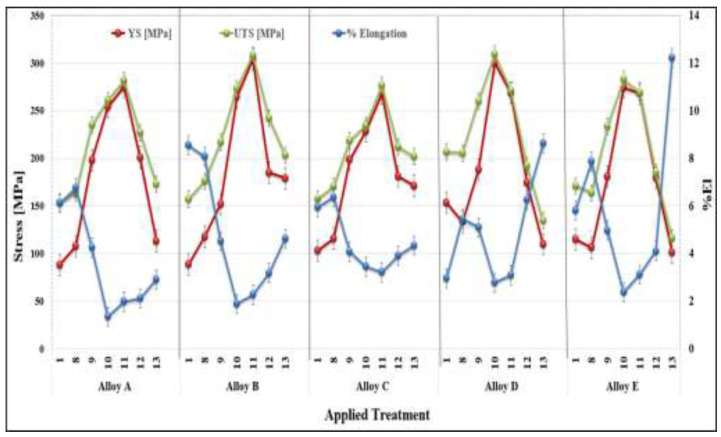
Variation in the tensile properties of the alloys tested at 250 °C as a function of the alloy composition and the applied heat treatment.

The tensile properties of the alloys A, B, C, D, and E when tested at 250 °C, using test bars in the as-cast and heat-treated conditions following solution heat treatment for 8 h, are presented in this section. Prior to testing, the test bars were kept in the testing chamber at 250 °C for thirty minutes to ensure a homogeneous temperature distribution throughout the bar before the test was carried out. The high temperature tensile properties (UTS, YS, and %El) of alloys A, B, C, D, and E are shown in Figure 10.

The five alloys achieved peak strength when T6 heat treatments were used (i.e., treatments 10 and 11) as the precipitates were fine, coherent, and displayed small inter-particle spacing so that the strength significantly increased. From Figure 10, it may be seen that alloys A, B, and C reached their peak strength with the heat treatment condition 11 as also presented in Table 5. When these tensile results are compared with those of the as-cast condition of each alloy, significant improvement in strength is noted.

When the T7 temper was applied (i.e., the heat treatments 12 and 13), the strength started to decrease, and the ductility started to increase. The reason behind that can be explained as, in the heat treatment condition 12, the aging temperature was further increased, which caused over-aging. In the heat treatment condition 13, the temperature as well as the aging time were increased, which caused further over-aging. As over-aging took place, the precipitates became coarser in size and lower in density, displaying large inter-particle distances as well. This facilitates dislocation motion that, in turn, has softening effects, which decrease the strength. Thus, when the castings were over-aged, the strength decreased, and the ductility increased.

**Figure 11:**
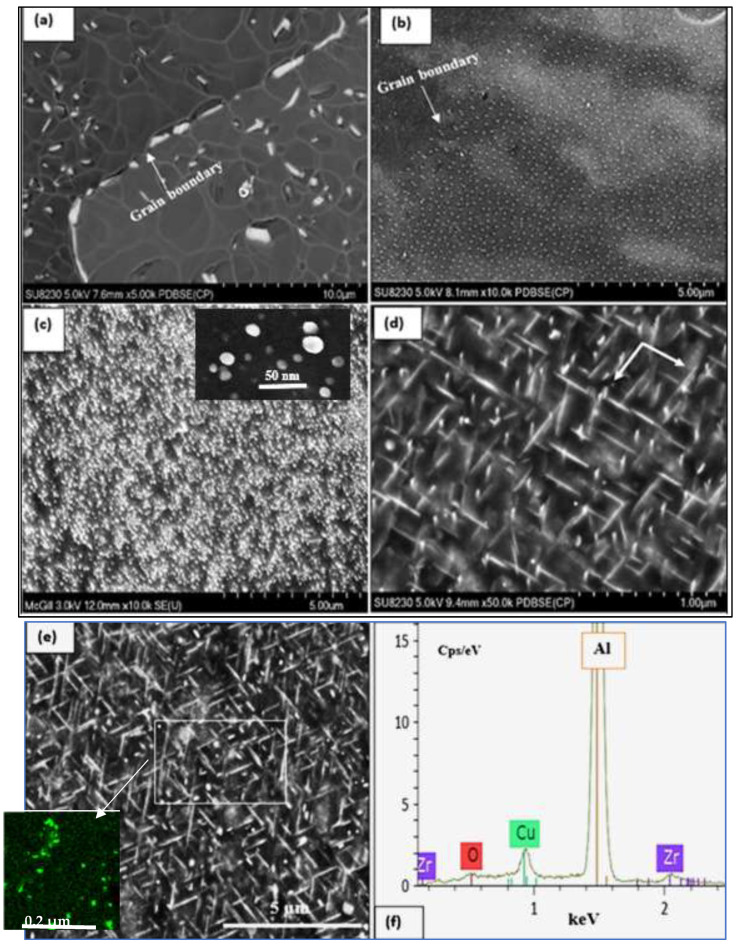
Precipitation of Al_2_Cu phase particles in alloy B: in the (**a**) as-cast and (**b**) SHT conditions; (**c**) aged 4 h at 180 °C (T6) and tested at 250 °C—inset shows almost complete sphericity of the precipitated particles; (**d**) aged 100 h at 250 °C following solutionizing at 520 °C for 4 h prior to testing at 250 °C; (**e**) electron image of alloy B in the T7 condition (**d**) tested at 250 °C—inset reveals Zr-rich precipitates; (**f**) EDS spectrum corresponding to the white rectangle in (**e**).

Figure 11a depicts the microstructure of the T200 alloy in the as-cast condition. In order to evaluate the effectiveness of the selected solutionizing treatment, i.e., time and temperature, the sample was aged at room temperature for one week. As can be seen in Figure 11a, a large amount of Al_2_Cu phase particles were precipitated during the T4 temper. Figure 11b exhibits the presence of some ultra-fine precipitated particles that may have occurred during the interval between the solution heat treatment (SHT) and examining the sample. With the high amount of Cu in the T200 alloy, a high-volume fraction of ultra-fine particles is to be expected when the alloy is aged at 180 °C (T6) as shown in Figure 11c. There is an explicit increase in their size and morphology from round particles to short rods arranged in two perpendicular directions, as displayed by the white arrows in Figure 11d, following aging for 100 h (T7). Using heat treatment changed that situation and enhanced the mechanical properties of the T200 alloys. Considering the testing temperature, Figure 11e illustrates the marked increase in the density of the precipitated Al_2_Cu when the alloy tensile bars were pulled to fracture at 250 °C. It should be mentioned here that, due to the high density of Al_2_Cu particles as displayed in Figure 11e, Al_3_Zr phase precipitation could not be traced. In addition, the work of Kipling et al. [18] was carried out at much higher temperatures in the range of 375–425 °C. The EDS spectrum, corresponding to the white rectangle in Figure 11e and presented in Figure 11f, reveals peaks due to the presence of Zr.

In general, improvement in the strength of the alloys is attributed to the solution heat treatment as well as the high solidification rate that followed. As with SHT, the maximum number of hardening solutes of Cu are retained in solid solution in the matrix forming a homogeneous supersaturated solid solution (SSSS) at elevated temperatures. When quenched or cooled rapidly, the SSSS formed during the solution treatment is preserved by means of rapid solidification to some lower temperature, usually near room temperature. This retains the solute atoms in solution and blocks them in their positions where they moved to at the high temperature during the SHT, which makes the casting ready for subsequent strengthening mechanisms [19,20,21,22].

### 3.3. Q-Charts

#### 3.3.1. Testing Temperature 25 °C

**Figure 12:**
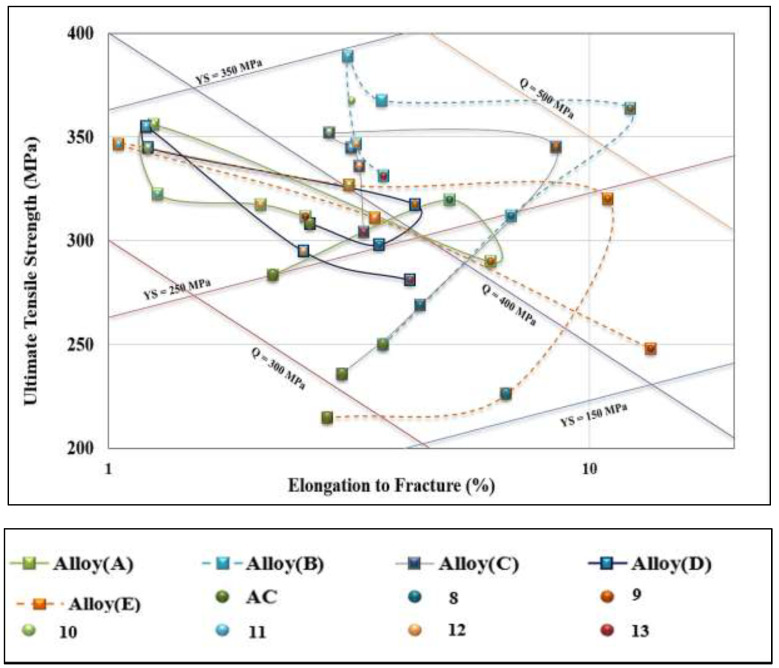
Quality chart showing the relationship between UTS and %El for the D and E alloys investigated in the as-cast and six heat treatment conditions (with SHT for 8 h).

Figure 12 presents the quality chart of the five studied alloys. The high Q and high PYS results would be located in the upper-right corner of the chart for alloys B, C, and E following T4 treatment (SHT for 8 h followed by warm-water quenching). Treatments 10 and 11 resulted in moving all the five alloys to the opposite corner of Figure 12, i.e., upper-left corner. Table 6 summarizes the composition and appropriate heat treatment for the T200 base alloy to achieve the best combination of *Q* and *PYS* values and each alloy that would result in the best Q and PYS values.

Although alloy B produced a Q value falling between alloys A and C, its corresponding PYS value is the highest among the three alloys. Thus, application of the T6 temper to alloy B may represent the optimum conditions for using alloy T200. Alloy B also showed higher values for both heat treatment conditions both without and with aging than the reference alloys D and E. In general, both alloys B and E exhibited a wide range of Q and PYS values compared to alloy D that represented the shortest cycle. It can be concluded, therefore, that alloy B in the T6 condition provides the optimum alloy composition/heat treatment condition for achieving the best tensile properties and alloy quality for the T200 alloy at room temperature. On the other hand, alloy E (broken red line) revealed the widest range of response to heat treatment in terms of Q levels (270 MPa–480 MPa) and PYS values (165 MPa–345 MPa).

**Figure 13:**
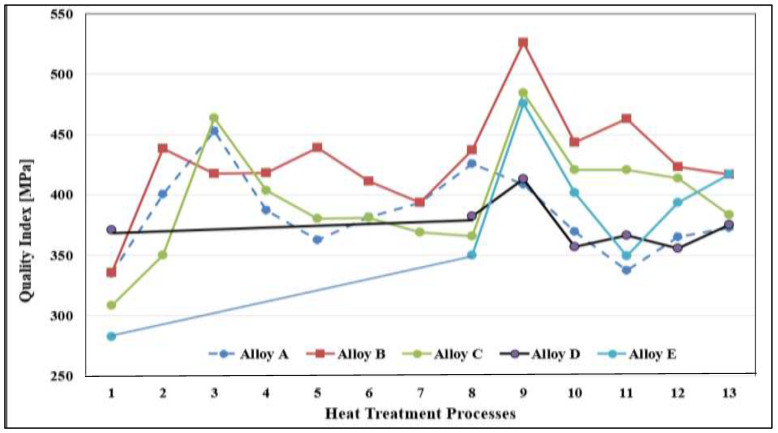
Quality values of alloys A, B, C, D, and E in the as-cast condition and all 13 heat treatment conditions used in this study.

Figure 13 presents a panel chart showing the Q-values corresponding to alloys A, B, C, D, and E for the 13 applied heat treatments including the as-cast condition. In the solution heat treatment condition (treatment #3), alloy C revealed the highest Q value above the commercial alloys. However, in the T6 temper, alloys C and E obtained very close values. In all, alloy B offered the highest Q levels over the whole heat treatment duration.

#### 3.3.2. Testing Temperature 250 °C

**Figure 14:**
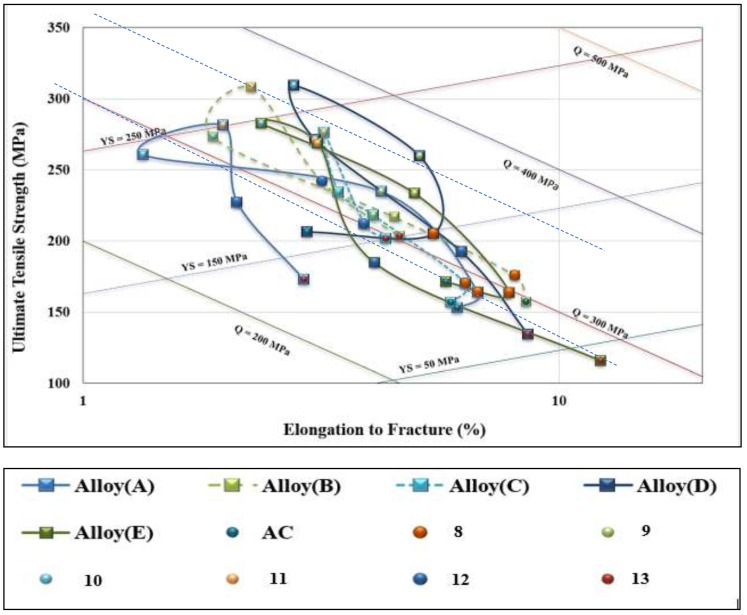
Quality chart showing the relationship between UTS and %El for the five alloys investigated in the as-cast and six heat treatment conditions with SHT for 8 h.

**Figure 15:**
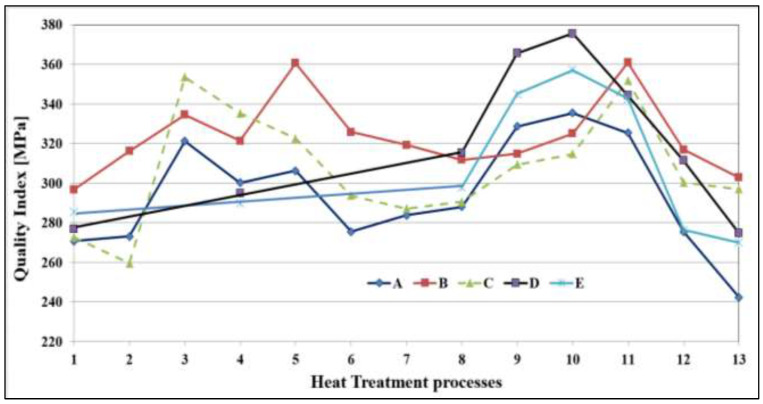
Quality Index-Heat Treatment relationships for the alloys tested at 250 °C.

Figure 14 shows a quality chart illustrating the relationship between UTS and %El for the five alloys in the as-cast and six heat treatment conditions tested at 250 °C. In general, all values fall in a narrow band around Q (290–350 MPa) as marked by the broken lines in Figure 14. The main difference appears in the PYS values, which vary between 50–270 MPa. For all alloys, over-aging (coded treatment 13) displayed values as low as Q = 250 MPa for alloy A, whereas alloy E showed the lowest PYS level of 40 MPa. In this case, as well, alloy E revealed the longest path between the as-cast and over-aging conditions due to the alloy heat treatment flexibility. Apparently, Q values of alloy B fall between alloy D (350 MPa) and alloy A (240 MPa). In comparing the Q and PYS values of alloy B with alloy E, alloy B shows slightly higher results. Therefore, it is reasonable to state that alloy B in the T6 temper may be considered as the optimum alloy composition/heat treatment condition. In comparison, as Figure 15 shows, alloy E exhibited Q values in the range 270 MPa–350 MPa and PYS levels in the range 20 MPa–250 MPa (Figure 14).

## 4. Fragtography

**Figure 16:**
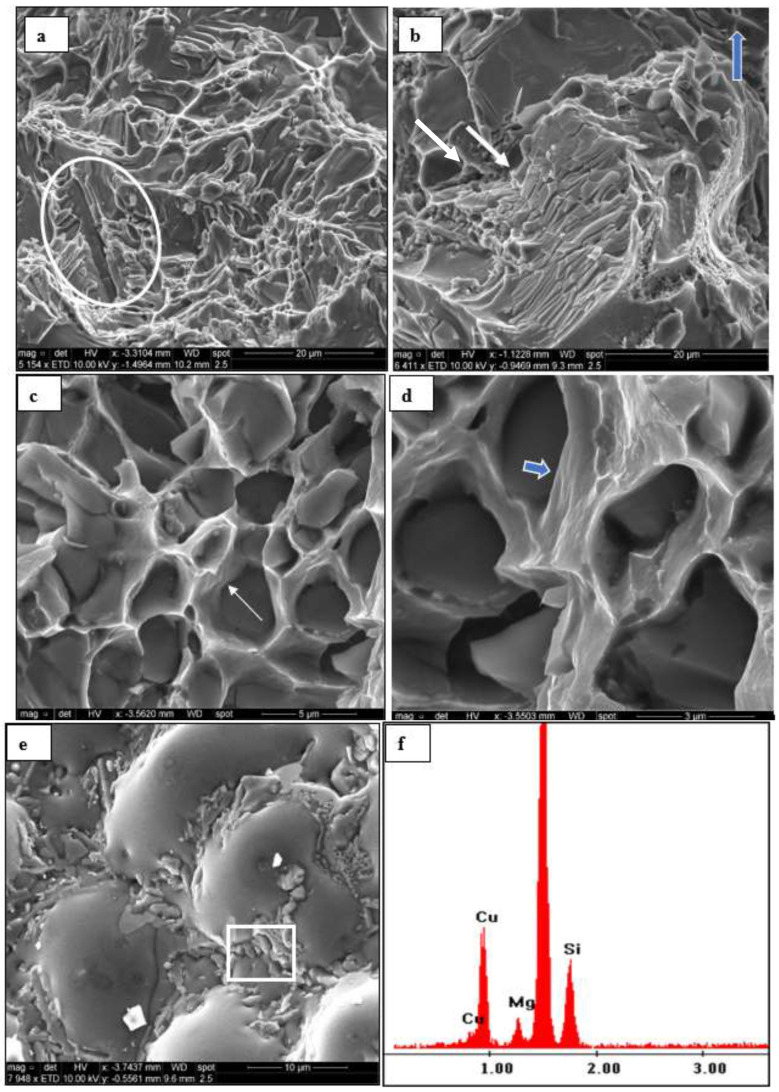
Secondary electron images showing the fracture surface of alloy B tested at 25 °C: (**a**) as-cast condition; (**b**) after T6 treatment; (**c**) after T7 treatment condition 13; (**d**) an enlarged micrograph of (**c**), (**e**) alloy D in the heat-treated condition 13; (**f**) EDS spectrum corresponding to (**e**). The blue arrow in (**b**) indicates the tensile direction.

In this section, the fracture behavior of alloy B will be discussed in terms of heat treatment and testing temperature. Figure 16a is the fracture surface of the alloy in the as-cast condition. Since the alloy does not practically have Si, the fracture occurs through the Fe-intermetallics, mainly α-Fe, which constituted the main intermetallic in this category of alloys (see white circle). The reported increase in the alloy strength following the T6 treatment (i.e., heat treatment condition 4) appeared in the fracture of the intermetallics over several parallel layers (see white arrows in Figure 16b) normal to the tensile axis (arrowed blue). Figure 16c represents the condition corresponding to the maximum attainable ductility of alloy B (heat treatment condition 13—Figure 4). As can be seen, the original as-cast structure is replaced by a network of deep dimples with slip marks on their walls as indicated by the white arrow. Figure 16d is an enlarged micrograph of Figure 16c highlighting the slip lines (see blue arrow). The fracture surface of the A319 alloy treated in the same condition 13 is displayed in Figure 16e for comparison, revealing the precipitation of coarse Al_2_Cu phase particles in the interdendritic region as confirmed from the associated EDS spectrum shown in Figure 16f, corresponding to the square area in Figure 16e, mixed with some Q-phase particles. Since the EDS is taken from a fracture surface, it is difficult to rely on the accuracy of the phase composition. 

**Figure 17:**
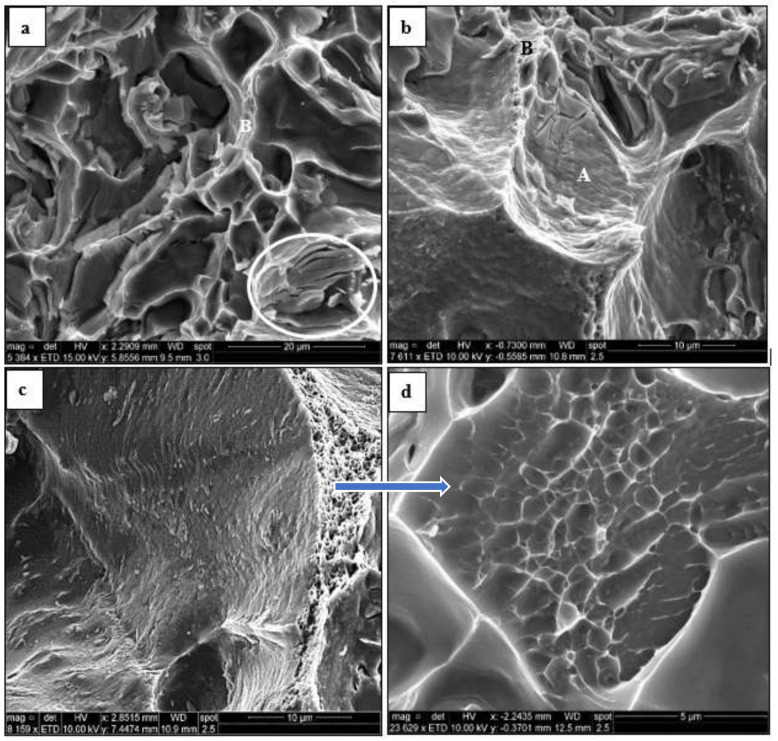
Secondary electron images of the fracture surface of alloy B tested at 250 °C: (**a**) T6 aged condition (180 °C/4h), (**b**–**d**) T7 condition (250 °C/100 h).

Figure 17 illustrates the variation in the fracture details of alloy B as a function of heat treatment when the tensile bars were tested at 250 °C. The fracture surface depicted in Figure 16a mainly revealed a dimple structure compared to that presented in Figure 17a for the same heat treatment but tested at 25 °C. Occasionally, some signs of brittle fracture similar to those marked by the circled area in Figure 17a were observed. Samples treated in the T7 condition with prolonged aging time (250 °C for 100 h) prior to pulling to fracture revealed two distinct areas marked A and B as shown in Figure 17b. Detail of the area marked A is shown in Figure 17c revealing a long series of slip lines covering the entire surface of the deformed dimple. Apparently, the gaps between two adjacent dimples—see the area marked B in Figure 17a—are composed of an ultra-fine dimple network (approximately 600 nm).

## 5. Conclusions

Based on the obtained results presented in this article, the following conclusions may be drawn:

1. Proper grain refining of the T200 alloy (alloy A) using TiBor in the amount of 0.15% Ti coupled with 0.28% Zr leads to the production of flexible heat-treatable castings free of hot-tearing defects.
2. Optimum heat treatment of this alloy is 4 h/520 °C 9SHT) followed by water quenching (~70 °C). Recommended artificial aging is 4 h at 180 °C regardless of the testing temperature.
3. Due to the high copper content in the T200 alloy, its tensile properties are superior to those obtained from the traditional A319 alloy.
4. Alloy B in the T6 condition is considered the optimum alloy composition/heat treatment condition for the T200 alloy. It resulted in the highest *UTS*, *YS*, *%El*, and Q-values compared with alloys A and C.
5. Alloy E (356 alloy) revealed the widest range of response to heat treatment in terms of *Q* levels (285 MPa–480 MPa) and *PYS* values (165 MPa–345 MPa) when tested at 25 °C. Testing at 250 °C resulted in *Q* values in the range of 270 MPa–350 MPa and *PYS* levels in the range of 20 MPa–250 MPa.
6. The presence of Ag in alloy C enhanced the alloy’s resistance to softening during the aging treatment.

## Figures and Tables

**Table 1:** Chemical composition of the as-received alloys for the present work.

Chemical Analysis (wt%)									
Alloy	Elements								
	Cu	Si	Fe	Mn	Mg	Ti	Zr	V	Al
T200	6.5	0.054	0.05	0.453	0.006	0.09	0.18	0.01	Balance
B319	3.32	7.97	0.418	0.245	0.266	0.131	-	-	Balance
A356	0.12	7.19	0.12	-	0.32	0.12	-	-	Balance

**Table 2:** Compositions of the five alloys used for this study.

Alloy Code	Composition
Alloy **A**	T200
Alloy **B**	T200 +0.15% Ti
Alloy **C**	T200 +0.15% Ti + 0.5%Ag
Alloy **D**	A319 + 0.15%Ti + 200 ppm Sr (0.02%)
Alloy **E**	A356 + 0.15%Ti + 200 ppm Sr (0.02%)

**Table 3:** Details of the heat treatment conditions for Alloys A, B, C, D, and E.

Heat Treatment Number	Heat Treatment Details
1	As cast
2	SHT for 4 h/air quenching
3	SHT for 4 h/water quenching
4	SHT for 4 h/water quenching + aging 1 (4 h @ 180 °C)
5	SHT for 4 h/water quenching + aging 2 (4 h @ 200 °C)
6	SHT for 4 h/water quenching +aging 3 (4 h @ 250 °C)
7	SHT for 4 h/water quenching + aging 4 (100 h @ 250 °C)
8	SHT for 8 h/air quenching
9	SHT for 8 h/water quenching
10	SHT for 8 h/water quenching + aging 1 (4 h @ 180 °C)
11	SHT for 8 h/water quenching + aging 2 (4 h @ 200 °C)
12	SHT for 8 h/water quenching + aging 3 (4 h @ 250 °C)
13	SHT for 8 h/water quenching + aging 4 (100 h @ 250 °C)

**Table 4:** Tensile properties of alloy A.

Condition	UTS [MPa]	YS [MPa]	% El [%]
As-cast	283.5	227.3	2.2

**Table 5:** Tensile properties of the five alloys following treatment 11 (T6 temper).

Alloy Code	UTS [MPa]	YS [MPa]	% Elongation
**A**	281	275	1.95
**B**	307	294	2.26
**C**	275	262	3.2
**D**	309	285	2.8
**E**	282	271	2.4

**Table 6:** Appropriate composition and heat treatment for the five alloys studied.

Alloy Code	Heat Treatment Code	Q (MPa)	PYS (MPa)
**A**	2	453	306
**B**	11	440	360
**C**	3	463	311
**D**	8	410	290
**E**	10	400	309

## Data Availability

Data will be made available upon request.
